# Maternal-infant relationship quality and risk of obesity at age 5.5 years in a national US cohort

**DOI:** 10.1186/1471-2431-14-54

**Published:** 2014-02-24

**Authors:** Sarah E Anderson, Stanley Lemeshow, Robert C Whitaker

**Affiliations:** 1 Division of Epidemiology, The Ohio State University College of Public Health, 336 Cunz Hall, 1841 Neil Avenue, Columbus, Ohio 43210, USA; 2 Division of Biostatistics, The Ohio State University College of Public Health, 204 Cunz Hall, 1841 Neil Avenue, Columbus, Ohio 43210, USA; 3 Departments of Public Health and Pediatrics and Center for Obesity Research and Education, Temple University, 1801 N Broad Street, Philadelphia, Pennsylvania 19122, USA

**Keywords:** Mother-child relationship, Parent–child interactions, Maternal sensitivity, Obesity, Cohort studies, Infancy, Parenting, Body mass index

## Abstract

**Background:**

Poor quality relationships between mothers and toddlers have been associated with higher risk for childhood obesity, but few prospective studies of obesity have assessed maternal-child relationship quality in infancy. In addition it is not known whether the increased risk is associated with the mother’s or the child’s contribution to the relationship quality.

**Methods:**

We analyzed data (n = 5650) from the Early Childhood Longitudinal Study, Birth Cohort, a national study of U.S. children born in 2001 and followed until they entered kindergarten. At 9 months of age, the Nursing Child Assessment Teaching Scale (NCATS) was used to assess the quality of observed playtime interactions between mothers and infants, yielding separate scores for maternal and infant behaviors. Obesity (BMI ≥95th percentile) at age 5.5 years was based on measured weight and height.

**Results:**

The prevalence (95% confidence interval) of obesity at 5.5 years of age was higher among children in the lowest quartile of maternal NCATS score (20.2% [95% CI: 17.2%, 23.2%]) than in the highest quartile (13.9% [11.3%, 16.5%]), but maternal NCATS score was not significantly associated with obesity after adjustment for race/ethnicity, maternal education and household income. The prevalence of obesity at 5.5 years of age was similar among children in the lowest quartile of infant NCATS score (17.4% [14.4%, 20.3%]) and in the highest quartile (17.6% 14.4%, 20.8%]), and was not changed with covariate adjustment.

**Conclusions:**

Maternal-infant relationship quality, assessed by direct observation at 9 months of age in a national sample, was not associated with an increased risk of obesity at age 5.5 years after controlling for sociodemographic characteristics.

## Background

Whether the quality of parent–child interactions is related to children’s obesity risk is not a new research question [1,2], but systematic investigation of this topic, particularly in population studies, has been limited [3]. We have provided evidence from two national studies that maternal-child interactions characterized by an insecure pattern of attachment and low levels of maternal sensitivity are associated with higher risk for obesity [4,5]. Others have also identified low levels of maternal warmth and sensitivity as risk factors for obesity [6-9]. Maternal sensitivity is one contributor to the quality of maternal-child relationships and increases the likelihood that a child will develop a secure pattern of attachment [10,11].

Evolving with time, the maternal-child relationship is shaped by the behavior and responses of both the mother and the child [12-14]. Just as parenting behavior can affect children, children’s behavior also influences parenting [13,15]. The construct of mutually responsive orientation has been developed to describe the positive, reciprocal, and synchronous interactions that are characteristic of high-quality parent-child relationships [16]. There is innate variability in infant reactivity, predictability, responsiveness, mood, and activity level [17,18], and the contributions of children’s temperamental attributes to early childhood obesity prevention are being examined [19]. The need to include dyadic conceptualizations and measurements of parent-child interaction in obesity research has been recognized [3], but there have been few studies of childhood obesity that have examined the contributions of both the parent and the child to parent-child relationship quality.

Infancy is an important time period to study the quality of maternal-child interactions relative to obesity risk because it is during this time period that the limbic areas of the brain governing stress response and emotion regulation are rapidly developing in relationship to the social environment [20]. These areas of the brain are also involved in regulation of energy balance which can be disrupted by an exaggerated stress response [21]. In two longitudinal studies we have shown that those toddlers who have poorer quality relationships with their mothers were at greater risk of later obesity [4,5]. In the current study, we used a large national sample to determine whether poorer quality interactions between mothers and infants increased the risk of childhood obesity, and to examine whether any increased risk was due to the behaviors of the infants, the mothers, or to both participants in the dyadic interaction.

## Methods

### Early Childhood Longitudinal Study, Birth Cohort (ECLS-B)

We analyzed data collected in the Early Childhood Longitudinal Study, Birth Cohort (ECLS-B), a study conducted by the National Center for Education Statistics (NCES) [22]. The NCES ethics review board approved the data collection, and parents provided written informed consent. Analyses reported here were conducted at The Ohio State University under a restricted-use data agreement. This agreement requires that we report unweighted sample sizes by rounding to the nearest 50.

The ECLS-B was designed to be representative of children born in the United States in 2001. The complex sampling design of the study has been previously described [22], and is briefly summarized here. A clustered list-frame design was used to select a probability sample of 14000 U.S. births in 2001. Children were excluded from the study if they were born to mothers under 15 years of age or if, before 9 months of age, infants had died or were adopted. When the children were 9 months of age, the final study cohort of 10700 was formed, and additional assessments took place when children were 24 months old (n = 9850) and approximately 4.5 years old (n = 8900). For budgetary reasons, an 85% random subsample (n = 7700) was selected for inclusion in the follow up at age 5.5 years, and 6950 children were assessed at that time [23]. Data were collected during visits to the children’s homes, and included a computer-assisted personal interview with the child’s mother (or, in a small number of cases, the father or other guardian), and direct measurements and observations of children and mothers.

### Maternal-infant interaction

During the 9-month home visits, the Nursing Child Assessment Teaching Scale (NCATS) was used to assess interactions between mothers and infants [24]. The NCATS is a standardized tool for assessing interactions between young children (0 to 36 months of age) and their parent or caregiver, and it has been used widely in research and clinical settings [14,25]. The NCATS is scored for 73 aspects of mother-infant interaction (50 items for the maternal score, 23 for the infant score). Higher scores indicate a higher quality (more adaptive and mutually responsive) pattern of interaction between mother and child. The maternal NCATS items assess maternal sensitivity to infant’s cues, response to infant’s distress, and fostering of infant’s social-emotional and cognitive growth [14]. The infant NCATS items assess the infant’s clarity of cues and infant’s responsiveness to the mother. To conduct the NCATS, mothers were shown a list of “activities” ordered by difficulty and asked to teach their infant the first activity on the list that he/she was not yet able to do; for example, “reach for a rattle, scribble on a piece of paper, stack 2 blocks on top of each other” [14]. The ECLS-B field interviewers videotaped the interaction between mother and infant, and the videotapes were coded in a central location by trained coders who had demonstrated reliable coding [25]. The internal consistency (Cronbach’s alpha) of the maternal and infant NCATS scores in ECLS-B were, respectively, 0.68 and 0.62 [25].

### Obesity

At 5.5 years of age, children’s height and weight were measured in the home using a standardized protocol. Height was measured with a portable stadiometer and weight was measured with a digital scale (both instruments from Seca [Hanover, MD]) while children were wearing light clothing and no shoes [26]. Measurements were taken three times, and the average value of the two closest measurements was used to calculate the child’s body mass index (BMI = kg/m^2^). We categorized children as obese if they had a sex-specific BMI-for-age at or above the 95th percentile of the U.S. Centers for Disease Control and Prevention growth reference [27].

### Covariates

Additional variables were considered for inclusion in our analyses based on their established relationship with obesity and possible relationship with maternal-infant interaction. Birth weight was obtained from birth certificate records. At the 9-month interview with the mother, each child’s age, gender, and race/ethnicity were obtained, and mothers were asked whether they had ever breastfed the child, the age of the child at weaning, and when solid foods were introduced [28]. Data were not available to determine the practice or duration of exclusive breastfeeding. Mothers were classified as smokers if they reported smoking cigarettes at the time of the 9-month interview. Maternal BMI was calculated from measured weight and self-reported height. We used two variables, maternal education and household income-to-poverty ratio, to estimate socioeconomic status (SES); both were derived from responses to the 9-month interview. The income-to-poverty ratio was calculated relative to 2002 U.S. poverty levels [29].

### Statistical analysis

Our analyses included the 5650 children who had data available on NCATS at 9 months and measured height and weight at 5.5 years. Of the 6950 children in ECLS-B that were assessed at 5.5 years, we excluded 1350 children—1200 children who did not participate in the NCATS assessment or whose videotape was not codable and 150 children who were missing height or weight at 5.5 years. We applied ECLS-B sampling weights, which include adjustments for disproportionate sampling, undercoverage of the target population, and nonresponse at each wave of data collection [30]. Variance estimates that account for the complex sample design were calculated using Jackknife replicate weights [23,31] as implemented in the survey procedures in SAS [32].

We created quartiles for the maternal and infant NCATS scores. We examined covariates relative to the prevalence of being obese at 5.5 years, in the lowest quartile of maternal NCATS score, and in the lowest quartile of infant NCATS score, and we used the Rao-Scott design-corrected *Χ*^2^ to test for group differences. We used logistic regression [33] to estimate odds ratios (ORs) and 95% confidence intervals (CIs) for childhood obesity comparing increasing quartiles of the maternal NCATS score and infant NCATS score to the lowest quartile. We assessed the functional form of the association between childhood obesity and NCATS scores using LOWESS smoothed scatterplots and the method of fractional polynomials [34]. Both maternal and infant NCATS scores were linear in the logit. We used logistic regression [33] to estimate the association of unit differences in maternal NCATS score or infant NCATS score, each modeled as a continuous variable, with odds for childhood obesity. Our analyses were conducted in steps; first unadjusted models were estimated, then models were adjusted for increasing numbers of covariates: 1) child level covariates (age, sex, birth weight, infant feeding), 2) maternal covariates (BMI and smoking) and 3) all covariates including race/ethnicity and SES. We present analyses with and without adjustment for SES because these variables may be confounding variables or may be part of a causal chain or pathway to obesity that also involves the quality of the maternal-infant relationship [4,35,36].

## Results

When compared to the analytic sample (n = 5650), children who were excluded for missing information (n = 1350) did not differ by sex (*P* = .26) but were more likely to have mothers who had not finished high school (24.9% vs. 17.7%, *P* < .001), to have an income-to-poverty ratio below 1.85 (54.7% vs. 46.5%, *P* = .01), and to be of Hispanic ethnicity (29.8% vs. 22.9%, *P* = .002). The prevalence of obesity at 5.5 years was 17.3% in the analytic sample and 19.4% among the 1200 children missing the NCATS (*P* = .25).

Maternal NCATS scores ranged from 15 to 49 with a mean (standard deviation [SD]) of 34.7 (4.5) and 25th, 50th, and 75th percentile values of 31.7, 34.8, and 37.8, respectively. Infant NCATS scores ranged from 7 to 23 with mean (SD) of 15.5 (2.7) and 25th, 50th, and 75th percentile values of 13.7, 15.7, and 17.0. The correlation of maternal to infant scores was r = .24. All of the maternal and child characteristics we examined, except for child sex and age at introduction of solid foods, were statistically significantly related to maternal NCATS score and to obesity at 5.5 years (Table 1). However, none of these same characteristics were associated with infant NCATS score (Table 1).

**Table 1:** Participant characteristics in relation to lowest quartile maternal and infant NCATS score and child obesity

	**N**^**a **^**(%)**	**Lowest quartile maternal NCATS**	**Lowest quartile infant NCATS**	**Obesity at 5.5 y**^**c**^
		**% (95% CI)**	**% (95% CI)**	**% (95% CI)**
Total	5650	23.3 (21.3 – 25.4)	23.1 (21.7 – 24.5)	17.3 (15.9 – 18.7)
Sex				
Female	2800 (49.3)	22.6 (20.2 – 25.1)	22.3 (20.5 – 24.1)	16.3 (14.2 – 18.3)
Male	2850 (50.7)	24.0 (21.6 – 26.4)	23.9 (21.6 – 26.2)	18.4 (16.2 – 20.5)
***P*-value**^**b**^		.69	.66	.17
Birth weight				
<1500 grams	550 (1.2)	29.4 (24.9 – 34.0)	31.1 (26.2 – 36.1)	8.8 (6.0 – 11.7)
1500 – <2500 grams	850 (6.1)	26.4 (22.5 – 30.3)	27.6 (23.7 – 31.6)	12.3 (9.3 – 15.2)
2500 – < 3000 grams	1050 (16.8)	27.0 (22.7 – 31.2)	26.0 (21.6 – 30.3)	13.2 (10.3 – 16.1)
3000 – < 3500 grams	1650 (37.9)	23.4 (20.3 – 26.5)	23.1 (20.7 – 25.5)	16.2 (13.7 – 18.7)
3500 – < 4000 grams	1150 (28.1)	21.7 (18.4 – 24.9)	20.0 (17.3 – 22.7)	18.8 (16.1 – 21.6)
≥ 4000 grams	350 (9.9)	19.1 (13.3 – 24.9)	23.2 (18.2 – 28.1)	28.9 (23.4 – 34.4)
***P*-value**^**b**^		.009	.15	<.0001
Duration of breastfeeding				
≥6 months	700 (13.5)	17.3 (13.0 – 21.6)	19.5 (15.3 – 23.6)	13.1 (10.0 – 16.2)
2 to <6 months	1350 (23.3)	25.0 (21.5 – 28.4)	24.8 (21.6 – 28.0)	17.3 (14.3 – 20.3)
<2 months	1800 (33.8)	21.0 (18.1 – 23.9)	22.9 (20.6 – 25.2)	15.8 (13.5 – 18.1)
Never	1700 (29.5)	27.6 (24.1 – 31.1)	23.8 (21.0 – 26.6)	21.0 (17.7 – 24.4)
***P*-value**^**b**^		<.0001	.69	.005
Age introduced solid foods				
≥6 months	1800 (26.2)	23.9 (19.8 – 28.0)	24.6 (21.5 – 27.7)	17.0 (14.7 – 19.3)
4-5 months	2700 (50.4)	22.6 (20.0 – 25.1)	22.8 (20.6 – 24.9)	16.2 (14.0 – 18.4)
0-3 months	1150 (23.4)	24.3 (20.5 – 28.2)	22.1 (18.9 – 25.4)	20.1 (17.0 – 23.1)
***P*-value**^**b**^		.14	.77	.09
Maternal BMI^d^				
<18.5 kg/m^2^	300 (4.5)	19.2 (12.4 – 26.0)	17.8 (12.6 – 23.0)	10.1 (4.7 – 15.4)
18.5 - <25 kg/m^2^	2250 (39.3)	21.8 (18.5 – 25.0)	23.0 (20.3 – 25.6)	9.3 (7.3 – 11.2)
25 - <30 kg/m^2^	1400 (28.1)	23.8 (20.5 – 27.1)	20.9 (17.7 – 24.1)	18.0 (15.5 – 20.4)
≥30 kg/m^2^	1550 (28.1)	24.6 (21.4 – 27.7)	25.8 (22.8 – 28.8)	28.9 (25.7 – 32.1)
***P*-value**^**b**^		.002	.40	<.0001
Maternal smoking status				
Nonsmoker	4600 (81.7)	22.4 (20.3 – 24.4)	23.0 (21.4 – 24.6)	16.6 (15.0 – 18.1)
Smoker	1050 (18.3)	27.7 (23.1 – 32.2)	23.8 (19.7 – 27.8)	20.9 (17.2 – 24.6)
***P*-value**^**b**^		<.0001	.90	.02
Racial-ethnic group				
White, non-Hispanic	2350 (55.1)	18.4 (15.7 – 21.1)	23.1 (20.9 – 25.4)	12.9 (11.2 – 14.5)
Black, non-Hispanic	950 (15.5)	24.3 (20.5 – 28.2)	20.4 (17.0 – 23.8)	21.2 (17.3 – 25.1)
Hispanic, any race	1000 (22.9)	34.4 (29.3 – 39.5)	25.2 (22.3 – 28.1)	24.1 (19.8 – 28.3)
Other race, non-Hispanic	1300 (6.4)	24.1 (19.4 – 28.8)	22.0 (17.8 – 26.2)	22.3 (16.9 – 27.6)
***P*-value**^**b**^		<.0001	.68	<.0001
Maternal education				
< High school	950 (17.7)	38.4 (33.1 – 43.8)	26.6 (22.8 – 30.5)	21.2 (17.6 – 24.9)
High school degree or GED	1450 (27.7)	29.3 (25.8 – 32.7)	23.2 (20.6 – 25.8)	20.6 (17.2 – 24.0)
Some college	1600 (29.1)	18.0 (15.1 – 21.0)	23.6 (20.8 – 26.4)	17.4 (14.9 – 20.0)
College graduate	1650 (25.4)	12.3 (9.9 – 14.7)	20.0 (17.4 – 22.6)	11.1 (8.8 – 13.3)
***P*-value**^**b**^		<.0001	.53	<.0001
Household income/poverty ratio				
< 0.50	650 (10.3)	38.3 (32.3 – 44.3)	23.9 (18.8 – 29.0)	20.8 (17.1 – 24.6)
0.50 to 0.99	700 (12.7)	29.9 (24.6 – 35.3)	25.6 (21.1 – 30.1)	22.6 (17.5 – 27.6)
1.00 to 1.85	1300 (23.5)	31.3 (26.9 – 35.7)	26.4 (23.0 – 29.8)	20.9 (17.8 – 24.0)
1.86 to 3.00	1550 (28.0)	17.6 (14.3 – 20.9)	21.0 (18.2 – 23.8)	14.8 (11.9 – 17.8)
>3.00	1450 (25.4)	13.0 (10.6 – 15.3)	20.8 (17.3 – 24.4)	12.8 (10.2 – 15.4)
***P*-value**^**b**^		<.0001	.07	<.0001

Restricted to children with information on NCATS at 9 months and weight-status at 5.5 years.

^a^N is unweighted and rounded to the nearest 50. Percents are weighted and 95% confidence intervals account for survey design. Percentages may not total 100% due to rounding.

^b^*P*-value from Rao-Scott design-adjusted *Χ*^2^ tests.

^c^BMI-for-age ≥95th percentile at age 5.5 years.

^d^Maternal BMI from reported height and measured weight at 9 month assessment. If maternal height was available but weight at the 9 month assessment was missing (n = 1000) then measured weight from the first available assessment was substituted [24 months (n = 700), 4.5 years (n = 250), 5.5 years (n = 50)].

Information missing for 150 individuals for maternal BMI, <100 individuals for breast feeding duration, and <50 individuals for birth weight, age introduced solid foods, maternal smoking status, and maternal education.

The prevalence of obesity at age 5.5 years was 20.2% (95% CI: 17.2% – 23.2%) among those children whose mother was in the lowest quartile of the maternal NCATS score compared to 13.9% (95% CI: 11.3% – 16.5%) among those whose mother was in the highest quartile. The prevalence of obesity was intermediate for the second and third quartiles of the maternal NCATS score, and the trend was statistically significant (Table 2). When modeled as a continuous variable in logistic regression, lower maternal NCATS score was related to higher odds of obesity at 5.5 years (*P* = .0012). Based on this unadjusted model, each 5 unit (approximately 1 SD) difference in maternal NCATS score at 9 months was associated with odds of obesity at 5.5 years that were 1.21 (95% CI: 1.08 – 1.36) times those of infants with mothers who had higher (worse) NCATS scores (Table 3). This estimate was little changed with adjustment for child age, sex, birth weight, and infant feeding variables, or with additional adjustment for maternal weight and smoking status (Table 3). However, with adjustment for all covariates the odds of obesity at 5.5 years associated with a 5 unit difference in maternal NCATS score was attenuated to 1.09 and was not statistically significant (95% CI: 0.95 – 1.24). This pattern of results was not changed by adjustment for infant NCATS score, and we observed no evidence that infant NCATS score modified associations between maternal NCATS score and obesity at 5.5 years (*P*-value for interaction = .99).

**Table 2:** Quartiles of maternal and infant NCATS scores at 9 months and obesity at 5.5 years

	**NCATS score range (min – max)**	**N (%)**^**a**^	**Obesity prevalence at 5.5 years (95% CI)**	**P trend**^**b**^	**Unadjusted OR (95% CI) for obesity at 5.5 years**
**Maternal**					
Q1 (low)	15.0 – 31.7	1400 (23.3)	20.2 (17.2 - 23.2)	.0012	1.57 (1.17 - 2.10)
Q2	31.8 – 34.7	1400 (24.6)	19.6 (16.5 - 22.6)		1.51 (1.11 - 2.05)
Q3	34.8 – 37.8	1450 (25.8)	16.1 (13.5 - 18.7)		1.19 (0.89 - 1.60)
Q4 (high)	37.9 – 49.0	1400 (26.3)	13.9 (11.3 - 16.5)		1.0 (referent)
**Infant**					
Q1 (low)	7.0 – 13.7	1450 (23.1)	17.4 (14.4 - 20.3)	.42	0.99 (0.74 - 1.32)
Q2	13.8 – 15.7	1450 (25.1)	20.0 (17.2 - 22.7)		1.17 (0.90 - 1.53)
Q3	15.8 – 17.0	1550 (28.5)	14.8 (12.5 - 17.1)		0.81 (0.61 - 1.09)
Q4 (high)	17.1 – 23.0	1200 (23.3)	17.6 (14.4 - 20.8)		1.0 (referent)

Restricted to children with information on NCATS at 9 months and weight-status at 5.5 years.

^a^N is unweighted and rounded to the nearest 50. Percents are weighted and 95% confidence intervals account for survey design. Percentages may not total 100% due to rounding.

^b^*P*-value from logistic regression models with NCATS score as a continuous variable; *P-*values for these trends were similar when quartiles were modeled.

**Table 3:** Logistic regression models of association between maternal NCATS score and obesity at age 5.5 years

**Model**	**N**	**Estimate**	**SE**	***P*-value**^**a**^	**OR (95% CI) for obesity associated with a 5 unit decrease in maternal NCATS score**^**b**^
Unadjusted	5650	0.038	0.012	.0012	1.21 (1.08 - 1.36)^c^
Adjusted for child covariates^d^	5500	0.039	0.013	.0017	1.22 (1.08 - 1.38)
Adjusted for child and maternal covariates^e^	5450	0.033	0.013	.01	1.18 (1.04 - 1.33)
Adjusted for all covariates^f^	5450	0.016	0.014	.23	1.09 (0.95 - 1.24)

^a^P from logistic regression modeling maternal NCATS as a continuous variable.

^b^Difference in maternal NCATS score of 5 units is approximately 1 SD.

^c^Results of unadjusted model were unchanged with exclusion of the 200 children who were missing information on ≥1 covariate.

^d^Covariates = age, gender, birth weight, duration of breast feeding, age when solid food introduced.

^e^Covariates = age, gender, birth weight, duration of breast feeding, age when solid food introduced, maternal BMI, maternal smoking.

^f^Covariates = age, gender, birth weight, duration of breast feeding, age when solid food introduced, maternal BMI, maternal smoking, race/ethnicity, maternal education, income-to-poverty ratio.

We observed no evidence of an association between infant NCATS scores and obesity at age 5.5 years irrespective of modeling approach. The prevalence (95% CI) of obesity was 17.4% (95% CI: 14.4% – 20.3%) in the lowest quartile of the infant NCATS and 17.6% (95% CI: 14.4% – 20.8%) in the highest quartile (Table 2). Modeled as a continuous variable in logistic regression analyses, lower scores on the infant NCATS were not associated with higher risk for obesity with or without covariate adjustment (*P*-values between .34 and .42).

Twenty of the items in the maternal NCATS score and twelve in the infant NCATS score are designated as “contingency items” because they are coded for behaviors made by one participant in the interaction (the mother or the child) in response to the other [14]. Consistent with what we observed for the full scores, the maternal contingency score was associated with higher risk for obesity at age 5.5 years in models that were not adjusted for SES, but not in models that adjusted for SES. The infant contingency score was not associated with obesity in either adjusted or unadjusted models.

## Discussion

We sought to assess the extent to which maternal and infant components of observed interactions between 9-month-old infants and their mothers were associated with obesity risk at 5.5 years of age in a large national cohort study. After controlling for SES, lower scores on the maternal component of the NCATS, which assesses maternal sensitivity, responsiveness, and fostering of infant emotional and cognitive growth, were not associated with an increased risk for obesity at 5.5 years of age. Our results showed no evidence that the infant’s contribution to the maternal-infant interaction affected the risk of obesity, nor did infant behavior modify the relationship between maternal behavior and children’s obesity risk.

Using observations of non-feeding interactions between mothers and infants, our goal was to examine separately the contributions of the infants’ and the mothers’ behaviors to the risk of childhood obesity. Prior research suggests that children’s self-regulation capacity is likely to be an important factor in the development of obesity [37-39]. Self-regulation, including emotion regulation, appears to be optimized by a parent–child relationship that has a mutually responsive orientation, meaning that interactions between parent and child are cooperative, coordinated, and harmonious, and involve the display of positive emotion [16,40,41]. Direct observation is the best method to assess whether the maternal-child relationship is mutually responsive [42]. The child’s behaviors, especially the child’s responsiveness to the mother, reflects more than the child’s temperament [43], and the importance of studying children’s contribution to the quality of maternal-child interaction in the context of obesity has been recognized [3]. Although the NCATS was not designed to assess mutually responsive orientation *per se*, it is based on direct observation and consists of separately coded behaviors for infants and mothers, including a subset of contingency items that assess the infant’s and mother’s responsiveness to one another.

Only a single study has examined the relationship between NCATS scores and childhood obesity [44]. Washington and colleagues recruited 200 low-income Mexican-American toddlers and their mothers at a WIC clinic in one Texas city. Half of the children were obese (BMI ≥95th percentile) and half were normal weight (BMI <85th percentile) and the NCATS was administered at two time points separated by six months [44]. Overall, there was no evidence that obese children had lower scores on the maternal or child NCATS, and some suggestion of *higher* scores for mothers of obese compared to normal weight toddlers [44]. However it is difficult to compare their findings to ours because of the small size and low sociodemographic heterogeneity of their sample.

We are not aware of additional studies of obesity and parent–child interaction that have directly assessed parent and child contributions to the quality of the relationship, but two studies have examined whether the relationship between maternal sensitivity and childhood obesity was modified by child temperament as reported by the parent. In a longitudinal analysis of 900 children studied between infancy and grade 6, Wu and colleagues found no relationship between infant temperament and the risk of being overweight or obese at school age (grades 1–6), but they did find that lower maternal sensitivity during infancy was associated with later weight status [9]. Although we did not find such a relationship, our study had a more socioeconomically diverse sample and involved a different measure of maternal interaction with the infant. Other prospective studies have shown that lower maternal warmth and sensitivity in interaction with toddlers [4,5], school-age children [6,8], and adolescents [7] is associated with a risk of later obesity. In a cross-sectional study of treatment-seeking obese youth (ages 8–16 years) in comparison to non-overweight controls, obesity was associated with low maternal warmth but only among children rated as temperamentally difficult [45].

### Limitations

Some ECLS-B participants were lost-to-follow-up or did not have NCATS data in infancy. This could have biased our findings and the direction of the bias is not possible to know. We controlled for infant birth weight and feeding, but we had no information on infant sleep quality or quantity. We did not have data on the interactions between fathers and infants; the quality of such interactions between fathers and infants may have affected children’s risk for obesity or modified how maternal-infant interaction was related to childhood obesity.

Although the NCATS is based on observed maternal-infant interaction and yields separate scores for maternal and infant behaviors, the measure has limitations. The internal consistency (Cronbach’s alpha) of the NCATS scores in ECLS-B were between 0.6 and 0.7, which is lower than the reliability values reported by others [14]. Our data were based on a single, brief interaction, which may not have been a typical one. Others have argued that the NCATS is more sensitive to cognitive than to affective components of mother-child interaction and contains items that may reflect cultural biases about parenting [46,47]. It is possible that the NCATS, as implemented in this study, did not adequately measure the emotional quality of the maternal interaction with the child, and this could be one reason we did not observe a stronger association between the quality of maternal interaction and children’s obesity risk.

In our study, maternal NCATS scores were related to race/ethnicity and sociodemographic characteristics, as had been seen in other studies [14]. In our analyses, we found that lower maternal NCATS scores were associated with obesity in models adjusted for the infant’s age, gender, birth weight, and feeding practices and the mother’s BMI and smoking status, but the association between maternal NCATS score and obesity was attenuated and no longer statistically significant with adjustment for race/ethnicity and sociodemographic characteristics—a pattern similar to what we have found previously [4]. We cannot distinguish with an observational study whether these results are due to a causal pathway going from these sociodemographic factors to the quality of the maternal-child relationship to obesity, or to confounding.

## Conclusions

In this longitudinal study of a large national sample, the risk of obesity at 5.5 years of age was not associated with maternal-infant relationship quality. The lack of association was clearer for the infant contribution to relationship quality than for the maternal contribution, which was significant before adjustment for SES. Our findings leave open the possibility that low levels of maternal sensitivity may increase risk for childhood obesity and suggest that further research focus on the mother’s not the infant’s, contribution to the quality of maternal-child relationship. This approach would be consistent with interventions that have demonstrated the potential to increase parental sensitivity [48], and in so doing have a beneficial impact on children’s weight [49].

## Abbreviations

BMI: Body mass index; CI: Confidence interval; ECLS-B: Early Childhood Longitudinal Study-Birth Cohort; NCATS: Nursing Child Assessment Teaching Scale; OR: Odds ratio; SD: Standard deviation; SES: Socioeconomic status.

## Competing interests

The authors declare that they have no competing interests.

## Authors’ contributions

SA and RW conceived the study and acquired funding. SA analyzed the data and drafted the manuscript. All authors were involved in interpretation of the data, critically revised the manuscript for important intellectual content, and read and approved the final manuscript.

## Pre-publication history

The pre-publication history for this paper can be accessed here:

http://www.biomedcentral.com/1471-2431/14/54/prepub
